# BRD4 is involved in viral exacerbation of chronic obstructive pulmonary disease

**DOI:** 10.1186/s12931-023-02348-y

**Published:** 2023-01-31

**Authors:** Yifei Duan, Siyi Zhou, Jianmiao Wang

**Affiliations:** Department of Respiratory and Critical Care Medicine, Tongji Hospital, Tongji Medical College, Huazhong University of Science and Technology, 1095 Jiefang Road, Wuhan, 430030 China

**Keywords:** Bromodomain protein 4 (BRD4), Chronic obstructive pulmonary disease, Exacerbation, Inflammation, Viral infection

## Abstract

**Background:**

Our previous studies have suggested that bromodomain protein 4 (BRD4) is increased in the lung of stable chronic obstructive pulmonary disease (COPD) patients, which has been shown to be involved in inflammatory responses. We investigated its role in the viral exacerbation of COPD.

**Methods:**

BRD4, interleukin (IL)-6 and IL-8 were measured in the blood and sputum of stable COPD patients and patients with viral exacerbation. Mice were exposed to cigarette smoke (CS) and/or infected with influenza virus as an in vivo model. BRD4, IL-6 and keratinocyte-derived chemokine (KC) were measured in the lung. BEAS-2B cells were treated with CS extract and/or influenza virus as an in vitro model. BRD4, IL-6 and IL-8 were measured in the cells and/or culture supernatant.

**Results:**

BRD4 was increased in COPD patients with viral exacerbation compared with those in stable condition and its expression was correlated with IL-6 and IL-8 expression. Inflammatory cells, IL-6, KC and BRD4 were synergistically induced in the lung of mice by viral infection and CS exposure, and the former three were decreased by JQ1 (BRD4 inhibitor) treatment. IL-6, IL-8 and BRD4 were significantly induced by CS extract and influenza virus in bronchial epithelial cells, and this upregulation was suppressed by knockdown of BRD4 expression.

**Conclusions:**

Our findings indicate that CS and viruses may synergistically induce IL-6 and IL-8 expression through their synergistic induction of BRD4 expression, which might contribute to the enhanced inflammatory response in the viral exacerbation of COPD.

**Supplementary Information:**

The online version contains supplementary material available at 10.1186/s12931-023-02348-y.

## Background

Chronic obstructive pulmonary disease (COPD) is a common respiratory disease, which is a leading cause of morbidity and mortality worldwide [1]. It is characterized by persistent respiratory symptoms and airflow limitation associated with an aberrant inflammatory response in the lung to noxious particles or gases. Cigarette smoke (CS) exposure is the most important cause of lung inflammation that induces tissue destruction and airway fibrosis, leading to the progressive airflow limitation [2].

Exacerbations of COPD are important events that accelerate the decline in lung function, contribute to disease progression, and increase the risk of death [3, 4]. Respiratory virus infections are associated with up to 40–60% of these exacerbations [5, 6]. As compared with nonviral exacerbations, viral exacerbations are associated with more severe symptoms, more frequent hospitalizations, and longer recovery periods [7, 8]. Many respiratory viruses have been shown to cause COPD exacerbations, among which the most common is rhinovirus. However influenza virus is more common in more severe exacerbations requiring hospitalization [9]. The molecular mechanisms that mediate the viral exacerbation in COPD have not been adequately defined.

Our previous studies have explored the effects of viral infection after CS exposure on the lung in mouse models, suggesting that CS and viruses interact in a manner to induce exaggerated lung inflammation [10, 11]. However the molecular mechanisms are complex, it has not been fully addressed whether these two challenges (CS and viruses) activate common pathways that can lead to synergistic changes in certain mediators and subsequently an enhanced inflammatory response in the lung.

Bromodomain protein 4 (BRD4) is a member of the bromodomain and extraterminal domain protein family, which plays an important role in the process of gene transcription [12, 13]. It can directly and indirectly regulate transcription both as a passive scaffold via its recruitment of vital transcription factors and as an active kinase that phosphorylates RNA polymerase [14, 15]. Both in vivo and in vitro studies have demonstrated that BRD4 inhibition significantly decreases the expression of proinflammatory cytokines, suggesting that BRD4 is involved in the inflammatory responses [16, 17]. Our previous studies have shown that BRD4 is increased and correlated with interleukin (IL)-8 expression levels in the lung of stable COPD patients, and in vitro studies have also suggested that BRD4 inhibition suppresses CS extract (CSE)-induced IL-8 expression in bronchial epithelial cells [18].

However, it is not clear whether BRD4 is involved in the viral exacerbation of COPD. In the present study, we explored BRD4 expression in the sputum and blood samples from stable COPD patients and COPD patients with viral exacerbation. We also utilized murine and cellular models to investigate the roles of BRD4 in the enhanced inflammatory response during viral infection after CS exposure.

## Methods

### Subjects

Stable COPD patients were recruited from the outpatient department of Tongji Hospital, Tongji Medical College, Huazhong University of Science and Technology, Wuhan, China, between 2020 and 2021. All patients were diagnosed with COPD according to the Global Initiative for Chronic Obstructive Lung Disease (GOLD) criteria [1]. COPD patients with viral exacerbation were recruited from the inpatient department of our hospital. An exacerbation of COPD was defined as an event characterized by increased dyspnea and/or cough and sputum that worsens in < 14 days. The detection of influenza virus nucleic acid in sputum was positive. Inclusion criteria: males aged between 40 to 80 years old with a history of at least 20 pack-years of smoking. Exclusion criteria: chronic respiratory diseases such as asthma, active tuberculosis, bronchiectasis and interstitial lung disease; cardiac, hepatic or renal failure; malignant diseases; autoimmune diseases; and current oral steroid therapy. In addition, COPD patients with viral exacerbation had a pulmonary function test report within 3 months before this exacerbation. The study was approved by the hospital ethics committees, and all subjects gave written informed consent.

### Pulmonary function tests

Forced vital capacity (FVC) and forced expiratory volume in the first second (FEV_1_) were obtained from the flow-volume curve using an appropriately calibrated spirometer (Jaeger, Wurzburg, Germany) before and 20 min after salbutamol inhalation. Three technically acceptable measurements were performed on each patient, and the highest value was selected and expressed as a percentage of reference values. The predicted FEV_1_ was calculated using the following prediction equations recommended by the American Thoracic Society/European Respiratory Society Task Force 2005 [19] (Predicted FEV_1_ = 4.30 × height in meters-0.029 × age-2.49).

### Sample collection

Heparinized peripheral venous blood and sputum samples were taken from the subjects. The plasma and blood cells were separated by centrifugation, and the blood cells were treated with red blood cell lysis buffer to obtain white blood cells and then stored appropriately until analyzed. Sputum induction with hypertonic saline was performed as previously described [20]. Sputum plugs were separated from sputum and dithiothreitol was used to disperse mucus. Sputum supernatant and cells were separated by centrifugation and stored appropriately until analyzed.

### Mouse models

C57BL/6 mice were purchased from the animal center of Tongji Medical College of Huazhong University of Science and Technology (Wuhan, China). All animal experiments were approved by the Institutional Animal Care and Use Committee of Tongji Medical College. Six to 8 weeks old male mice were exposed to room air (RA) or the smoke from nonfiltered 3R4F research cigarettes (University of Kentucky, Lexington, KY, USA) for 12 weeks using the smoking apparatus as previously described [21]. Mice received a half cigarette twice a day to allow for acclimation in the first week and received 1 cigarette twice a day thereafter. Mice were anesthetized and 1.5 × 10^3^ plaque forming units of A/PR8/34 (H1N1) influenza virus (Advanced Biotechnologies, Columbia, MD, USA) was administered via nasal aspiration in 50 μl of phosphate buffered saline (PBS) using techniques previously described [22]. BRD4 specific inhibitor JQ1 (MedChem Express, Shanghai, China) was delivered via intraperitoneal injection at a dosage of 50 mg/kg. Mice were injected once a week from the first day of CS exposure to the first day of virus infection.

### Bronchoalveolar lavage

Mice were sacrificed and the trachea was cannulated and perfused with two 0.75 ml aliquots of PBS. The cellular contents and bronchoalveolar lavage (BAL) fluid were separated by centrifugation. Total and differential leukocyte counts were determined and BAL fluid samples were stored at − 80 °C until analyzed.

### Immunohistofluorescence

The lung tissue was obtained from mice, fixed with 4% paraformaldehyde, embedded in paraffin and sliced. Lung slices were dewaxed and hydrated before heat-induced epitope retrieval was performed. After blocking, lung slices were incubated with BRD4 rabbit monoclonal antibody (Catalog No. A700-004, Bethyl, Montgomery, TX, USA) at 4 °C overnight. Slices were washed and incubated with DyLight 594-conjugated goat anti-rabbit IgG (Abbkine, Redlands, CA, USA) for 1 h at room temperature in the dark and then washed and coverslipped with Vectashield HardSet Mounting Medium with DAPI.

### Preparation of CS extract

CS extract (CSE) was prepared as described previously [11]. Briefly, CSE was freshly made by bubbling the smoke from two 3R4F research cigarettes without filter, at a rate of 1 cigarette/5 min, to a 50 ml conical tube containing 20 ml culture medium. The extract was filtered through a 0.22 μm filter and was regarded as 100% strength CSE.

### Cell culture

Human bronchial epithelial cells BEAS-2B cells (American Type Culture Collection, Manassas, VA, USA) were cultured in RPMI 1640 medium supplemented with 10% fetal bovine serum and 1% penicillin/streptomycin in a humidified incubator under 5% CO_2_ at 37 °C. Cells were stimulated with 5% CSE and/or infected with the influenza virus at the multiplicity of infection (MOI) of 0.5 for 24 h. In the transfection experiment, cells were transfected with BRD4 siRNA (RiboBio, Guangzhou, China) using Lipofectamine 3000 (Invitrogen, Carlsbad, CA, USA).

### Enzyme-linked immunosorbent assay

According to the product manual, enzyme-linked immunosorbent assay (ELISA) kits (R&D Systems, Minneapolis, MN, USA) were used to measure the protein levels of IL-6 and IL-8 in plasma, sputum supernatant and the culture supernatant of BEAS-2B cells. The protein levels of IL-6 and keratinocyte-derived chemokine (KC; a mouse homolog of IL-8) in the BAL fluid of mice were detected using ELISA kits (LiankeBio, Hangzhou, China) according to the product manual.

### Quantitative polymerase chain reaction

Total RNA was extracted from the white blood cells and sputum cells of patients, lung tissues of mice, and BEAS-2B cells. Reverse transcription was conducted with PrimeScript RT Reagent Kit (Takara, Shiga, Japan). Quantitative polymerase chain reaction (PCR) was performed using a Bio-Rad CFX Connect Real-Time System (Bio-Rad, Hercules, CA, USA) with SYBR Premix Ex Taq (Takara, Shiga, Japan) and the specific primers. The primer sequences were as follows: GAPDH (human, 5′-ACAACTTTGGTATCGTGGAAGG-3′ and 5′-GCCATCACGCCACAGTTTC-3′), BRD4 (human, 5′-ACCTCCAACCCTAACAAGCC-3′ and 5′-TTTCCATAGTGTCTTGAGCACC-3′), IL-6 (human, 5′-CTGCTGCCTTCCCTGCC-3′ and 5′-CCTCTTTGCTGCTTTCACACAT-3′), IL-8 (human, 5′-AAGAAACCACCGGAAGGAAC-3′ and 5′-ACTCCTTGGCAAAACTGCAC-3′), GAPDH (mouse, 5′-AGGTCGGTGTGAACGGATTTG-3′ and 5′-GGGGTCGTTGATGGCAACA-3′), BRD4 (mouse, 5′-CCTCCCAAATGTCTACAACGC-3′ and 5′-TGAGCAGATATTGCAGTTGGTT-3′), IL-6 (mouse, 5′-TAGTCCTTCCTACCCCAATTTCC-3′ and 5′-TTGGTCCTTAGCCACTCCTTC-3′), KC (mouse, 5′-ACTGCACCCAAACCGAAGTC-3′ and 5′-TGGGGACACCTTTTAGCATCTT-3′), M1 (viral, 5′-AAGACCAATCCTGTCACCTCTGA-3′ and 5′-CAAAGCGTCTACGCTGCAGTCC-3′). The relative mRNA expression was determined using the 2^−ΔΔCt^ methods with GAPDH as endogenous control.

### Statistical analysis

Data were expressed as mean ± SEM unless stated otherwise. D’Agostino and Pearson omnibus normality test was used to check whether the data conform to the normal distribution. Normally distributed data were assessed for significance by Student’s t-test or ANOVA as appropriate. Data that were not normally distributed were assessed for significance using the Mann–Whitney U-test or the Kruskal–Wallis test with Dunn’s posttest for multiple comparisons as appropriate. The correlations were analyzed by Pearson’s correlation. Prism version 8 (GraphPad) was used for data analysis. A two-sided p-value less than 0.05 was considered to be statistically significant.

## Results

### Characteristics of subjects

The characteristics of subjects are shown in Table 1. Totally, 54 subjects were recruited to this study including 25 stable COPD patients and 29 COPD patients with viral exacerbation. All of the subjects were male. There were no significant differences in age, body mass index and smoking index between stable patients and patients with exacerbation. There were also no significant differences in FEV_1_/FVC and FEV_1_%predicted (FEV_1_%pred) between these two groups.

**Table 1:** Clinical characteristics of subjects in this study

	sCOPD	AECOPD	*P* value
Subjects	25	29	
Age yrs	63.4 ± 1.6	65.9 ± 1.4	0.236
Male	25(100%)	29(100%)	
Smoking index p.y	39.4 ± 4.5	41.7 ± 3.5	0.695
BMI kg/m^2^	21.6 ± 0.7	22.7 ± 0.8	0.326
FEV_1_/FVC %	41.7 ± 2.8	41.5 ± 1.8	0.957
FEV_1_% pred	49.4 ± 4.4	45.6 ± 3.0	0.466

Values are numbers (%) or mean ± SEM

*sCOPD* stable chronic obstructive pulmonary disease, *AECOPD* acute exacerbation of COPD, *p.y* pack-yrs, *BMI* body mass index, *FEV*_*1*_ forced expiratory volume in 1 s, *FVC* forced vital capacity, *%pred* %predicted

### BRD4 expression is increased in COPD patients with viral exacerbation compared with those in stable condition

**Fig. 1:**
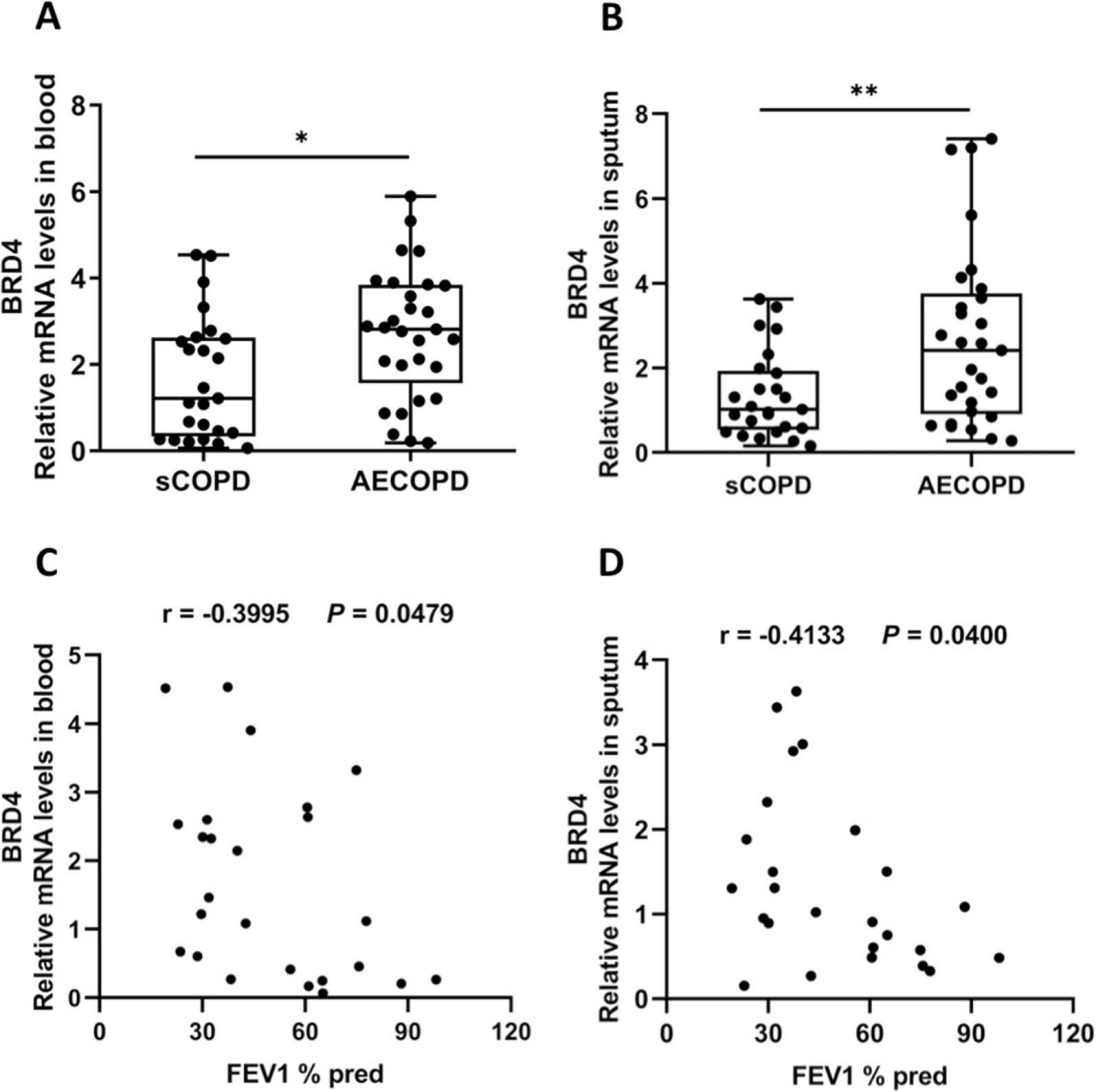
BRD4 expression is increased in COPD patients with viral exacerbation compared with those in stable condition. The relative mRNA levels (fold change) of BRD4 in the blood (**A**) and sputum (**B**) of stable COPD patients (sCOPD, n = 25) and COPD patients with viral exacerbation (AECOPD, n = 29) are shown. The correlation between the BRD4 expression levels in blood (**C**) and sputum (**D**) and forced expiratory volume in one second (FEV_1_) % predicted in stable COPD patients (n = 25) is shown. *P < 0.05, **P < 0.01

To study the expression of BRD4 in the blood and sputum of stable COPD patients and COPD patients with viral exacerbation, blood and sputum samples from the subjects were examined by quantitative PCR. We found that BRD4 expression was increased significantly both in the blood and in the sputum of COPD patients with viral exacerbation compared with stable COPD patients (Fig. 1A, B). We also analyzed the association between BRD4 expression and pulmonary function, and found that BRD4 levels both in the blood and in the sputum were significantly correlated with FEV_1_%pred in stable COPD patients (Fig. 1C, D).

### IL-6 and IL-8 expression is increased in COPD patients with viral exacerbation compared with those in stable condition

**Fig. 2:**
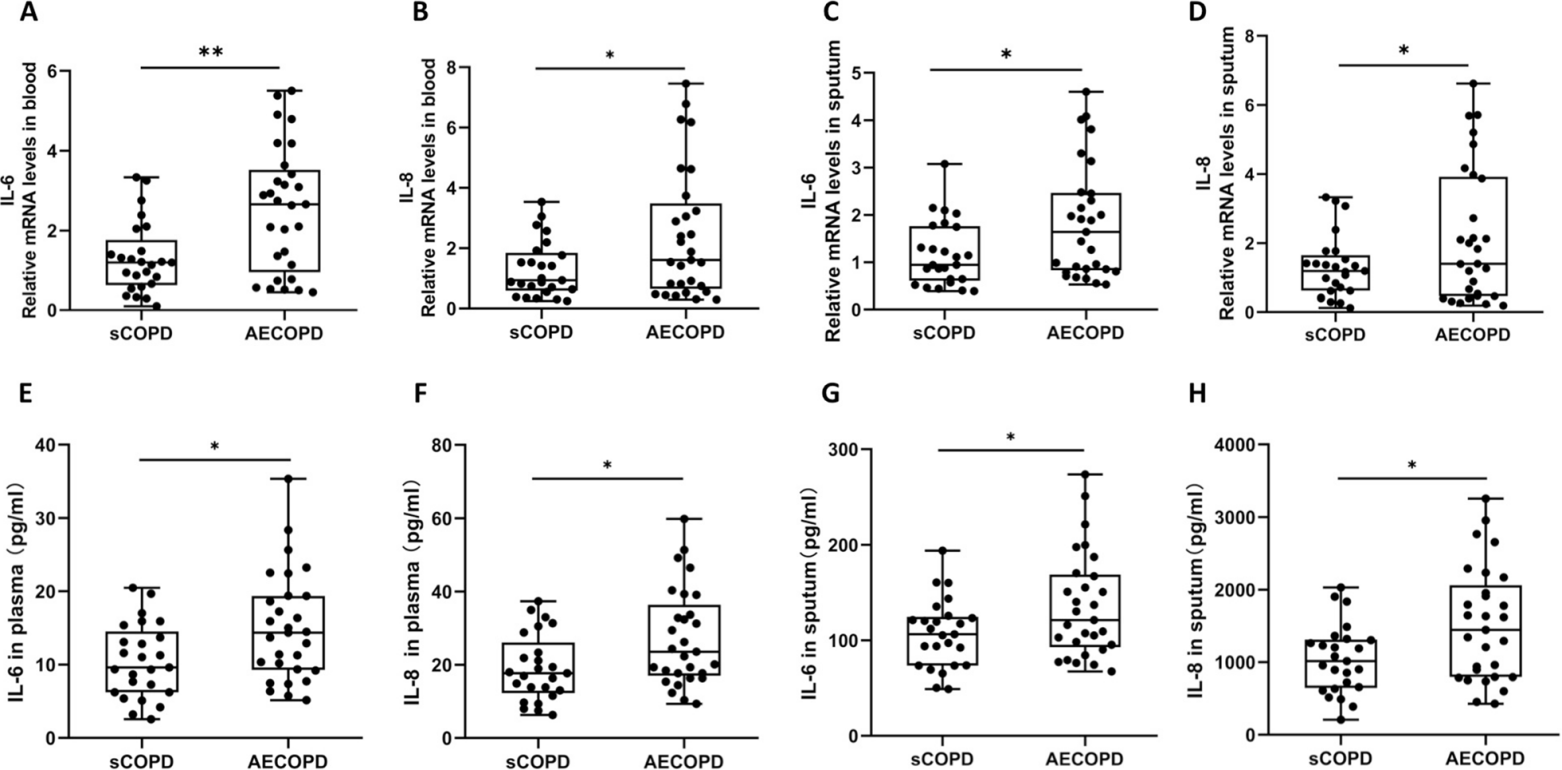
IL-6 and IL-8 expression is increased in COPD patients with viral exacerbation compared with those in stable condition. The relative mRNA levels (fold change) of IL-6 (**A**) and IL-8 (**B**) in the blood of stable COPD patients (sCOPD, n = 25) and COPD patients with viral exacerbation (AECOPD, n = 29) are shown. The relative mRNA levels (fold change) of IL-6 (**C**) and IL-8 (**D**) in the sputum of stable COPD patients (sCOPD, n = 25) and COPD patients with viral exacerbation (AECOPD, n = 29) are shown. The protein levels of IL-6 (**E**) and IL-8 (**F**) in the plasma of stable COPD patients (sCOPD, n = 25) and COPD patients with viral exacerbation (AECOPD, n = 29) are shown. The protein levels of IL-6 (**G**) and IL-8 (**H**) in the sputum of stable COPD patients (sCOPD, n = 25) and COPD patients with viral exacerbation (AECOPD, n = 29) are shown. *P < 0.05, **P < 0.01

To study the expression of IL-6 and IL-8 in stable COPD patients and COPD patients with viral exacerbation, IL-6 and IL-8 mRNA and protein levels were determined in the blood and sputum from the subjects by quantitative PCR and ELISA. We found that both IL-6 and IL-8 mRNA levels were increased significantly in the blood and sputum from COPD patients with viral exacerbation compared with stable COPD patients (Fig. 2A–D). IL-6 and IL-8 protein levels were also increased significantly in the blood and sputum from COPD patients with viral exacerbation compared with stable COPD patients (Fig. 2E–H).

### BRD4 expression is positively correlated with IL-6 and IL-8 expression in COPD patients

**Fig. 3:**
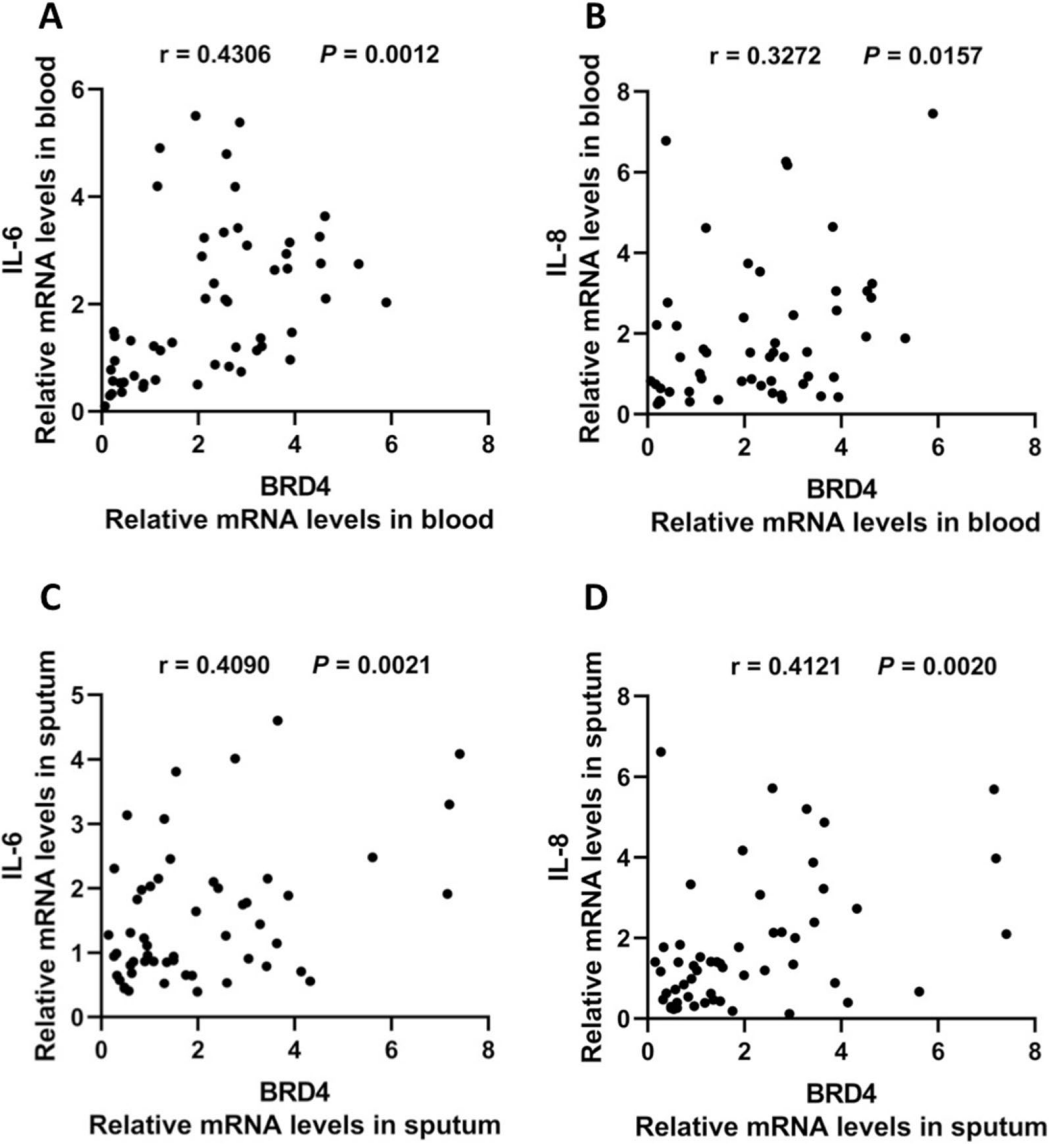
BRD4 expression is positively correlated with IL-6 and IL-8 expression in COPD patients. The correlation between the BRD4 expression levels and the expression levels of IL-6 (**A**) and IL-8 (**B**) in the blood of all COPD patients (n = 54) is shown. The correlation between the BRD4 expression levels and the expression levels of IL-6 (**C**) and IL-8 (**D**) in the sputum of all COPD patients (n = 54) is shown

To investigate the association between BRD4 expression and IL-6 and IL-8 levels, the correlations were analyzed by Pearson’s correlation. We found that BRD4 expression was positively correlated with IL-6 and IL-8 expression in the blood of all COPD patients (Fig. 3A, B). Similarly, BRD4 expression was positively correlated with IL-6 and IL-8 expression in the sputum of all COPD patients (Fig. 3C, D).

### IL-6 and KC expression in the lung of mice is synergistically induced by viral infection and CS exposure

**Fig. 4:**
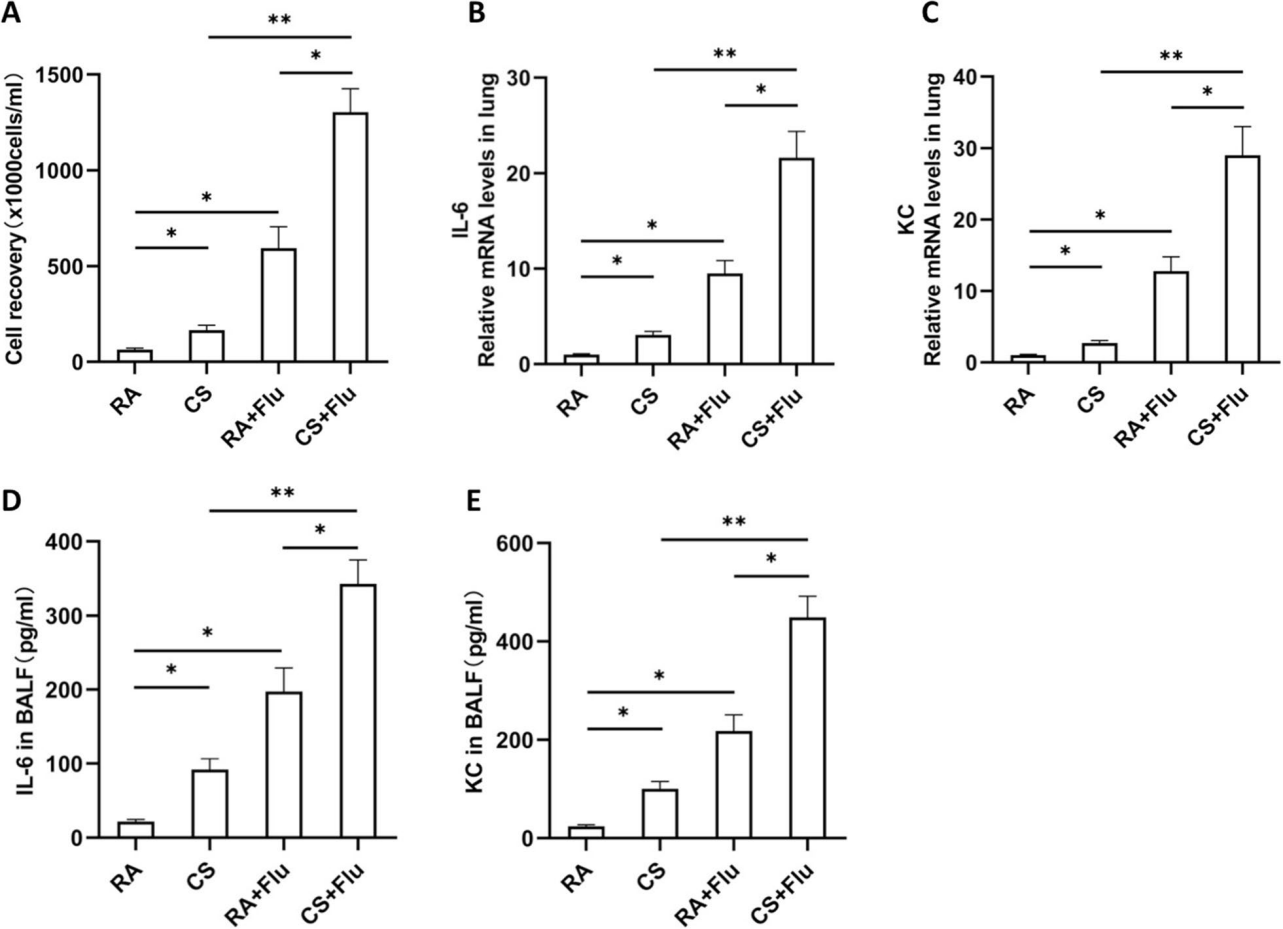
IL-6 and KC expression in the lung of mice is synergistically induced by viral infection and CS exposure. Mice were exposed to room air (RA) or cigarette smoke (CS) for 12 weeks and then infected with influenza virus or vehicle control. They were sacrificed and evaluated on day 7 after infection. The total leukocyte counts in bronchoalveolar lavage fluid (BALF) (**A**), the relative mRNA levels (fold change) of IL-6 (**B**) and KC (**C**) in the lung, the protein levels of IL-6 (**D**) and KC (**E**) in BALF are shown (n = 5 mice/group). RA + Flu: RA-exposed mice with influenza virus infection; CS + Flu: CS-exposed mice with influenza virus infection; *P < 0.05, **P < 0.01

To study the IL-6 and KC expression in the lung of CS-exposed mice after viral infection, mice were exposed to RA or CS for 12 weeks and then infected with influenza virus or vehicle control. They were sacrificed and evaluated on day 7 after infection. We found that the total leukocyte counts in BAL fluid of mice were synergistically induced by viral infection and CS exposure (Fig. 4A). The mRNA expression of IL-6 and KC in the lung of mice was also synergistically induced by viral infection and CS exposure (Fig. 4B, C). Similar changes were found in the protein levels of IL-6 and KC in the BAL fluid of mice (Fig. 4D, E).

### BRD4 expression in the lung of mice is synergistically induced by viral infection and CS exposure

**Fig. 5:**
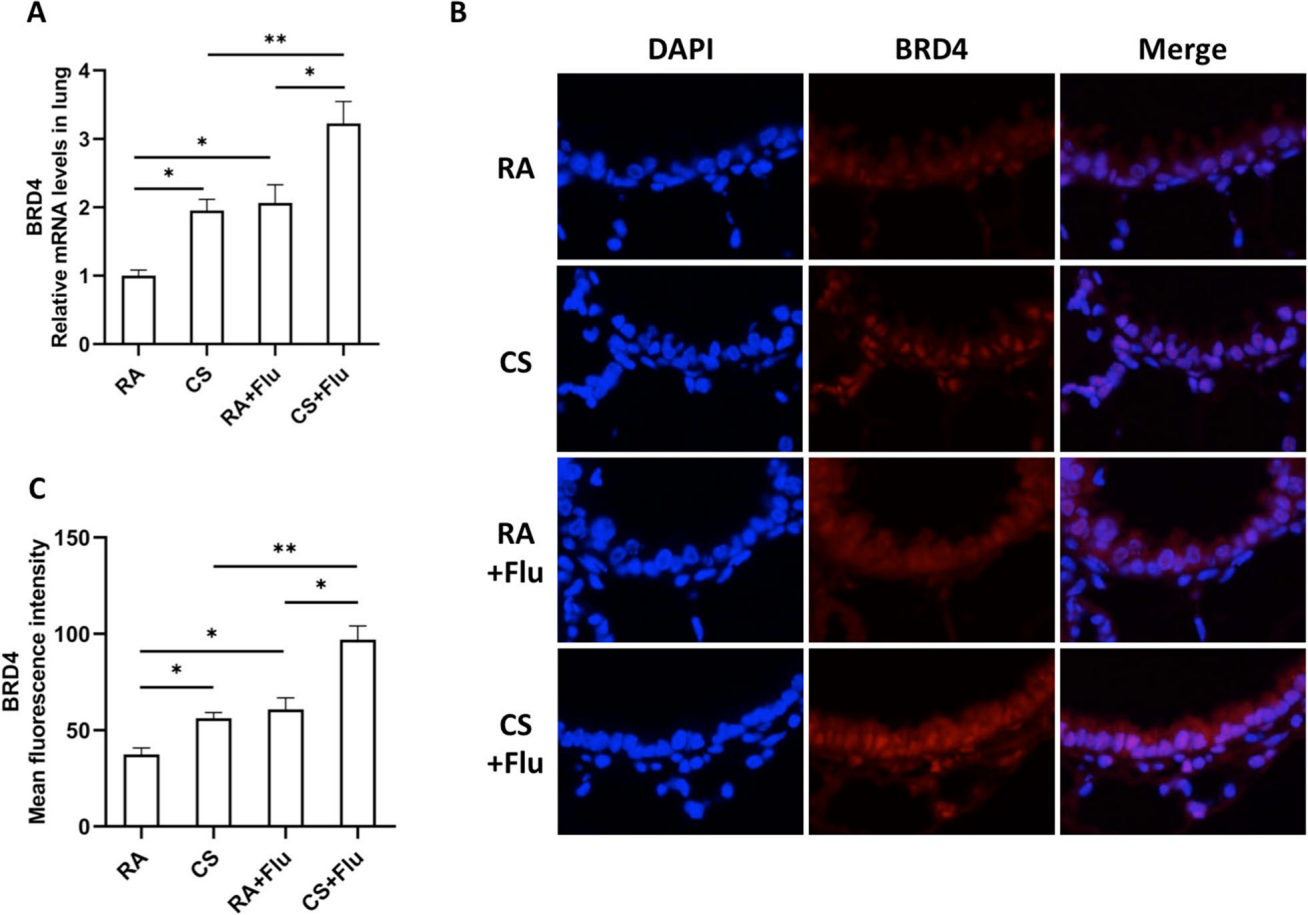
BRD4 expression in the lung of mice is synergistically induced by viral infection and CS exposure. Mice were exposed to room air (RA) or cigarette smoke (CS) for 12 weeks and then infected with influenza virus or vehicle control. They were sacrificed and evaluated on day 7 after infection. The relative mRNA levels (fold change) of BRD4 (**A**) in the lung are shown (n = 5 mice/group). The representative immunohistofluorescence stained lung sections (**B**) from mice are shown (BRD4: red, nuclear stain DAPI: blue). Original magnification, × 200. The mean fluorescence intensity values of BRD4 (**C**) are shown (n = 5 mice/group). RA + Flu: RA-exposed mice with influenza virus infection; CS + Flu: CS-exposed mice with influenza virus infection; *P < 0.05, **P < 0.01

To study the BRD4 expression in the lung of CS-exposed mice after viral infection, mice were exposed to RA or CS for 12 weeks and then infected with influenza virus or vehicle control. They were sacrificed and evaluated on day 7 after infection. The BRD4 expression measurements were performed on the same experiments as the ones used for measuring the cytokine expression. We found that the mRNA expression of BRD4 in the lung of mice was synergistically induced by viral infection and CS exposure (Fig. 5A). Immunohistofluorescence staining showed that BRD4 was strongly expressed in the bronchial epithelial cells after CS exposure and/or viral infection (Fig. 5B). Mean fluorescence intensity analysis showed that BRD4 expression was synergistically induced by viral infection and CS exposure (Fig. 5C).

### BRD4 inhibitor JQ1 suppresses IL-6 and KC expression in the lung of mice during viral infection after CS exposure

**Fig. 6:**
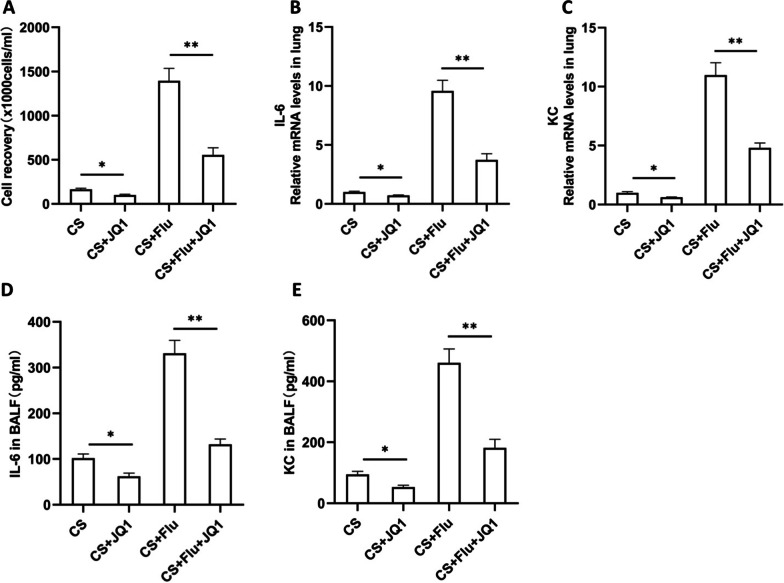
BRD4 inhibitor JQ1 suppresses IL-6 and KC expression in the lung of mice during viral infection after CS exposure. Mice were injected with BRD4 inhibitor JQ1 or vehicle control once a week from the first day of cigarette smoke (CS) exposure. After CS exposure for 12 weeks, mice were then infected with influenza virus or vehicle control. They were sacrificed and evaluated on day 7 after infection. The total leukocyte counts in bronchoalveolar lavage fluid (BALF) (**A**), the relative mRNA levels (fold change) of IL-6 (**B**) and KC (**C**) in the lung, the protein levels of IL-6 (**D**) and KC (**E**) in BALF are shown (n = 5 mice/group). CS + Flu: CS-exposed mice with influenza virus infection; *P < 0.05, **P < 0.01

To study the effect of BRD4 inhibition on the expression of IL-6 and KC, mice were injected with BRD4 specific inhibitor JQ1 or vehicle control once a week from the first day of CS exposure. After CS exposure for 12 weeks, mice were then infected with influenza virus or vehicle control. They were sacrificed and evaluated on day 7 after infection. We found that the total leukocyte counts in BAL fluid were decreased significantly by JQ1 treatment (Fig. 6A). The mRNA expression of IL-6 and KC in the lung of mice was decreased significantly by JQ1 treatment (Fig. 6B, C). The protein levels of IL-6 and KC in BAL fluid were also decreased significantly by JQ1 treatment (Fig. 6D, E).

### IL-6 and IL-8 are significantly induced by CSE and virus and are suppressed by knockdown of BRD4 in bronchial epithelial cells

**Fig. 7:**
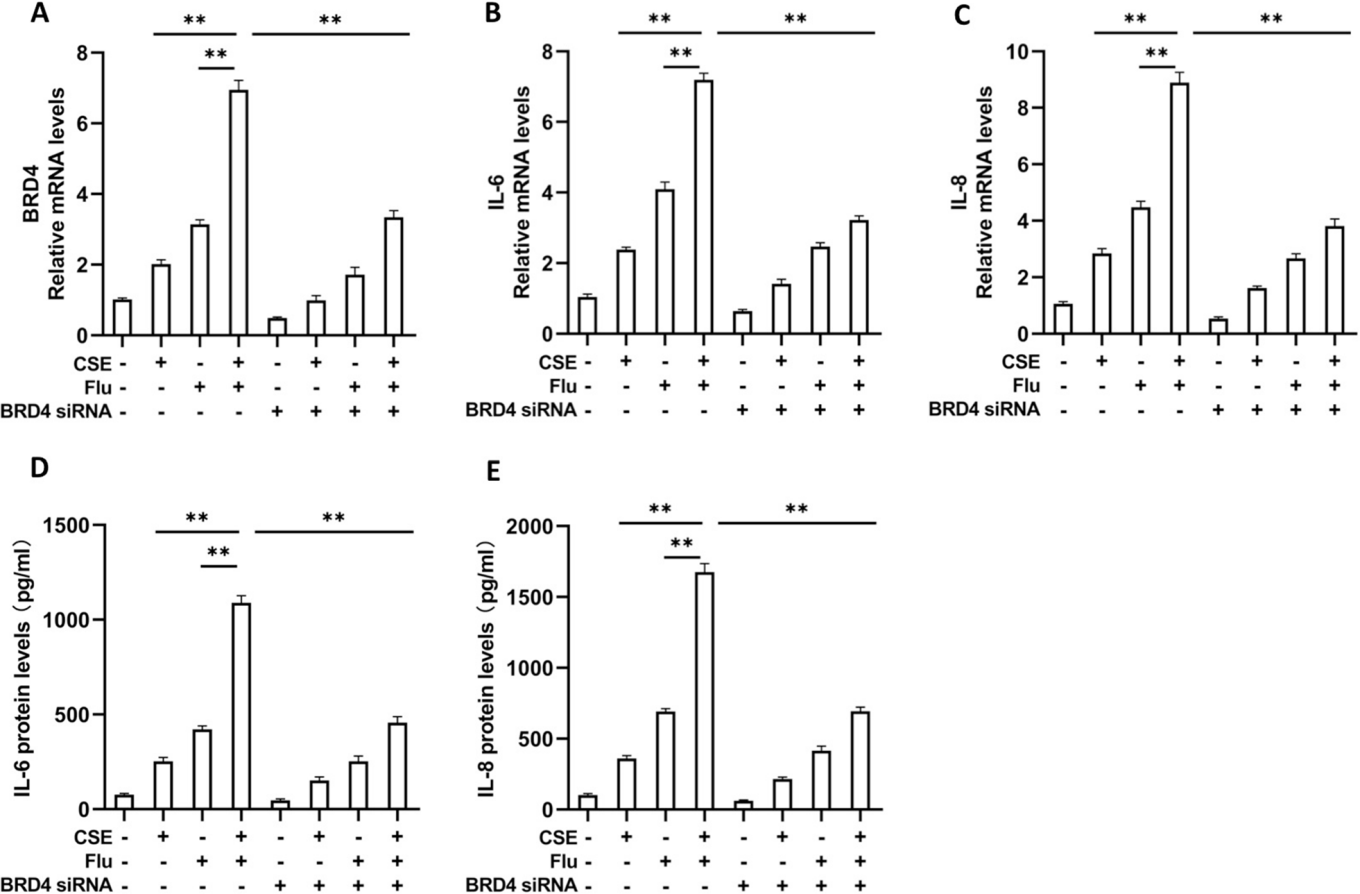
IL-6 and IL-8 are significantly induced by CSE and virus and are suppressed by knockdown of BRD4 in bronchial epithelial cells. BEAS-2B cells were treated with CSE and/or infected with influenza virus after transfection with BRD4 siRNA. The relative mRNA levels (fold change) of BRD4 (**A**), IL-6 (**B**), and IL-8 (**C**) in BEAS-2B cells are shown. The protein levels of IL-6 (**D**) and IL-8 (**E**) in cell culture supernatant are shown. The data are representative of at least three independent experiments. **P < 0.01

To further study the effect of BRD4 on the expression of IL-6 and IL-8 after viral infection and CS exposure, BEAS-2B cells were cultured and treated with CSE and/or influenza virus as an in vitro model. BRD4 expression was significantly induced by CSE plus virus in BEAS-2B cells, and this upregulation was suppressed by transfection of BRD4 siRNA (Fig. 7A). The mRNA expression of IL-6 and IL-8 in BEAS-2B cells was significantly induced by CSE plus virus, and this upregulation was suppressed by knockdown of BRD4 expression (Fig. 7B, C). The protein levels of IL-6 and IL-8 in the culture supernatant were also significantly induced by CSE plus virus, and this upregulation was suppressed by knockdown of BRD4 expression. (Fig. 7D, E). We also measured the viral RNA levels in the influenza virus-treated cells using quantitative PCR and found that CSE did not significantly affect the replication of influenza virus in these cells (Additional file 1: Fig. S1).

## Discussion

COPD exacerbations negatively impact health status, disease progression and mortality [23]. When associated with viral infections, exacerbations are often more severe, last longer and precipitate more hospitalizations [7, 8]. During exacerbations there is evidence of increased airway inflammation. In the present study, we show that inflammatory cytokines including IL-6 and IL-8 expression levels are increased significantly in the blood and sputum from COPD patients with viral exacerbation compared with stable patients. Using mouse models, our previous studies have shown that viral infection after CS exposure induces exaggerated lung inflammation [10, 11], suggesting that CS and viruses might activate common pathways that lead to synergistic changes in certain mediators and subsequently an enhanced inflammatory response. In the present study, we mainly explored the roles of BRD4 in this exaggerated inflammation.

BRD4 has been widely studied in cancer, cardiovascular, metabolic, autoimmune diseases [24]. It plays an important role in inflammation by regulating the expression of inflammatory genes [25]. Our previous studies have suggested that BRD4 is increased and is associated with lung inflammation in stable COPD patients [18]. It has been shown that BRD4 mediates lung inflammation in response to respiratory syncytial virus [26]. In the present study, we show that BRD4 is increased significantly in the blood and sputum from COPD patients with viral exacerbation compared with stable patients, and is positively correlated with IL-6 and IL-8 expression. These results imply that BRD4 might be involved in the enhanced inflammatory response in the viral exacerbation of COPD.

Consistent with our previous findings, we show that inflammatory cells in the BAL fluid of mice are synergistically induced during viral infection after CS exposure. In this mouse model, we also show that inflammatory cytokines including IL-6 and KC in the lung of mice are synergistically increased during viral infection after CS exposure. More importantly, BRD4 expression in the lung of mice is synergistically induced during viral infection after CS exposure. Immunohistofluorescence indicates that BRD4 expression is significantly increased in the bronchial epithelial cells of the mouse lung after viral infection or CS exposure. These two challenges synergistically induce BRD4 expression in the bronchial epithelial cells. Further, our intervention experiments show that the total leukocyte counts in BAL fluid and the expression of IL-6 and KC in the lung of mice are significantly decreased by BRD4 inhibitor JQ1 treatment during viral infection after CS exposure. These results suggest that BRD4 is necessary for IL-6 and KC expression and may play an important role in the enhanced inflammatory response.

In our in vitro experimental study, we show that BRD4 expression is significantly induced by CSE and influenza virus in bronchial epithelial cells. IL-6 and IL-8 expression in bronchial epithelial cells is also significantly induced by CSE and influenza virus, and this upregulation is suppressed by knockdown of BRD4 expression. Previous studies have explored the effects of viral infection after CS exposure on the lung in mouse models, which have demonstrated that CS and viruses interact in a manner to induce exaggerated lung inflammation [27–29]. Many of these studies have focused on the immune cells such as natural killer cells [28, 29]. However, lung inflammation in COPD also involves activation of structural cells such as airway epithelial cells which produce inflammatory mediators including IL-6 and IL-8 after being activated [30–32]. Our results suggest that CS and viruses may synergistically induce IL-6 and IL-8 expression in bronchial epithelial cells through their synergistic induction of BRD4 expression, which might contribute to the enhanced inflammatory response in the viral exacerbation of COPD.

Preclinical studies have demonstrated the benefit of BRD4 inhibition in a variety of diseases including malignant and inflammatory conditions [33–35]. However, the inhibitors have been most widely studied in the context of cancer. On the basis of several promising preclinical studies in hematological and solid malignancies, these drugs are being evaluated in clinical trials across the world. With the accumulation of relevant research, BRD4 is also becoming a biological target for drug development for the treatment of airway inflammation and relevant lung diseases.

In addition, in the present study, we show that BRD4 is also increased in the blood of COPD patients with viral exacerbation and its expression is positively correlated with IL-6 and IL-8 expression in the blood of COPD patients. The possible underlying mechanism is not explored, and further animal and cellular studies are needed to elucidate it in our future work.

In summary, we report that BRD4 is increased in COPD patients with viral exacerbation and its expression is correlated with IL-6 and IL-8 expression; Inflammatory cells, IL-6, KC and BRD4 are synergistically induced in the lung of mice by viral infection and CS exposure, and the former three are decreased by JQ1 treatment; IL-6, IL-8 and BRD4 are significantly induced by CSE and influenza virus in bronchial epithelial cells, and this upregulation is suppressed by BRD4 knockdown. Our findings indicate that CS and viruses may synergistically induce IL-6 and IL-8 expression through their synergistic induction of BRD4 expression, which might contribute to the enhanced inflammatory response in the viral exacerbation of COPD. BRD4 is expected to be an anti-inflammatory therapeutic target for this disease.

## Supplementary Information

**Additional file 1: Fig. S1.** The viral RNA levels in influenza virus-infected BEAS-2B cells with different treatments.

## Data Availability

Not applicable.

## Abbreviations

BAL: Bronchoalveolar lavage
BRD4: Bromodomain protein 4
COPD: Chronic obstructive pulmonary disease
CS: Cigarette smoke
CSE: CS extract
ELISA: Enzyme-linked immunosorbent assay
FEV_1_: Forced expiratory volume in one second
FVC: Forced vital capacity
GOLD: Global Initiative for Chronic Obstructive Lung Disease
IL: Interleukin
KC: Keratinocyte-derived chemokine
MOI: Multiplicity of infection
PBS: Phosphate buffered saline
PCR: Polymerase chain reaction
RA: Room air
