# Supplementary material for: BRD4 is involved in viral exacerbation of chronic obstructive pulmonary disease
Source: Respir Res. 2023 Jan 31;24:37. doi: 10.1186/s12931-023-02348-y (PMC9887738; doi:10.1186/s12931-023-02348-y)
Supplement: Supplementary file 1 — Additional file 1: Fig. S1. The viral RNA levels in influenza virus-infected BEAS-2B cells with different treatments. [file 12931_2023_2348_MOESM1_ESM.pdf]

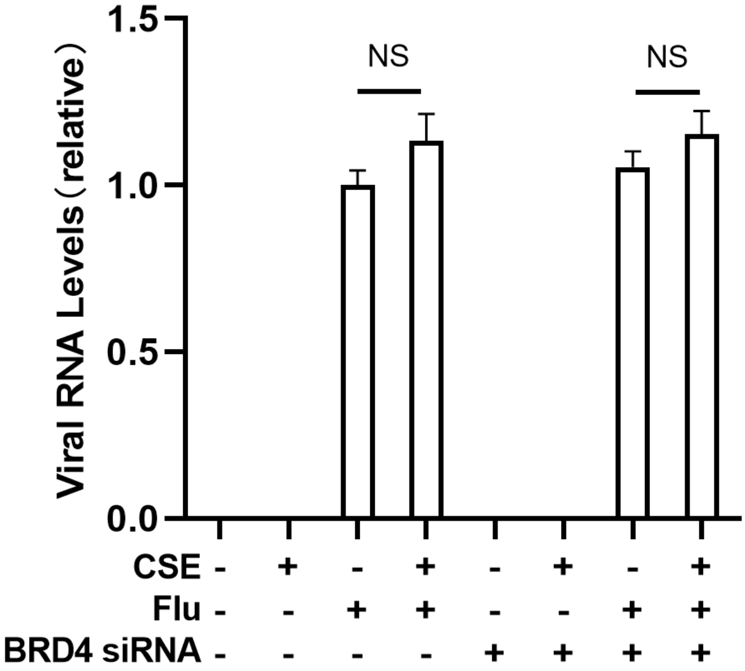

Figure S1. The viral RNA levels in influenza virus-infected BEAS-2B cells with different treatments. BEAS-2B cells were treated with CSE and/or infected with influenza virus after transfection with BRD4 siRNA. The relative viral RNA levels (fold change) of influenza virus in BEAS-2B cells are shown. The data are representative of at least three independent experiments. NS, nonsignificant.
